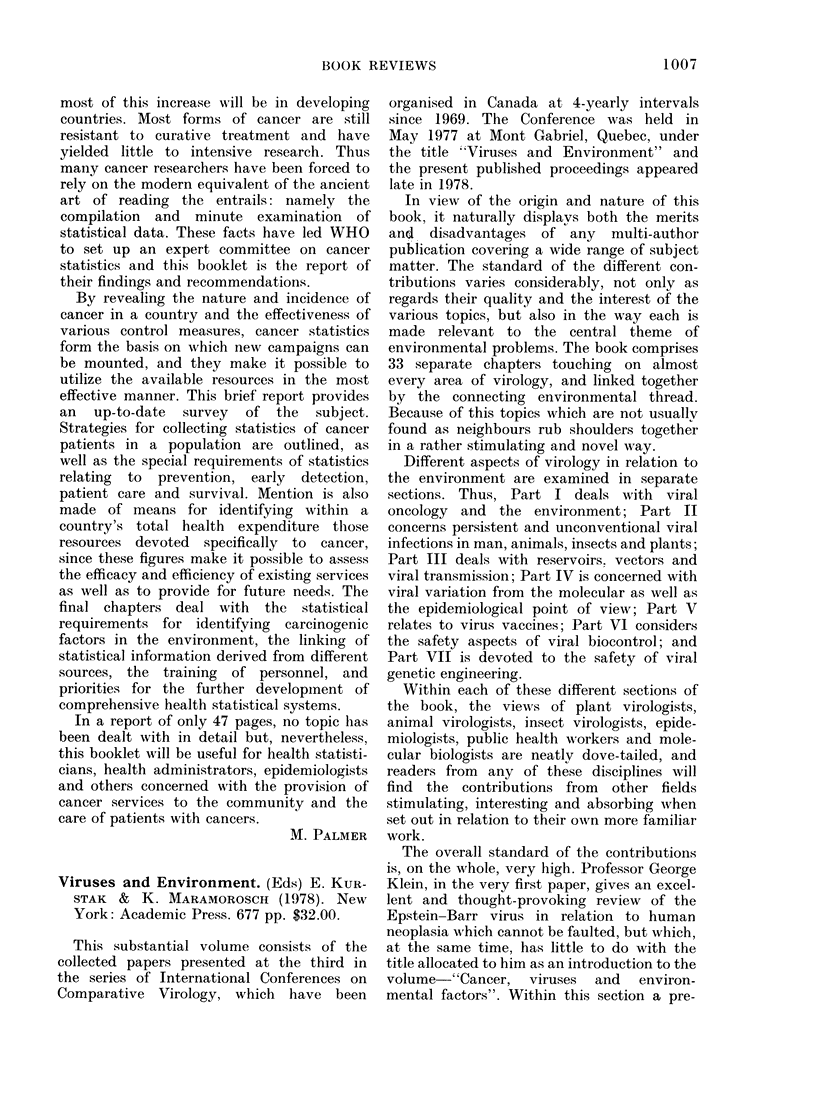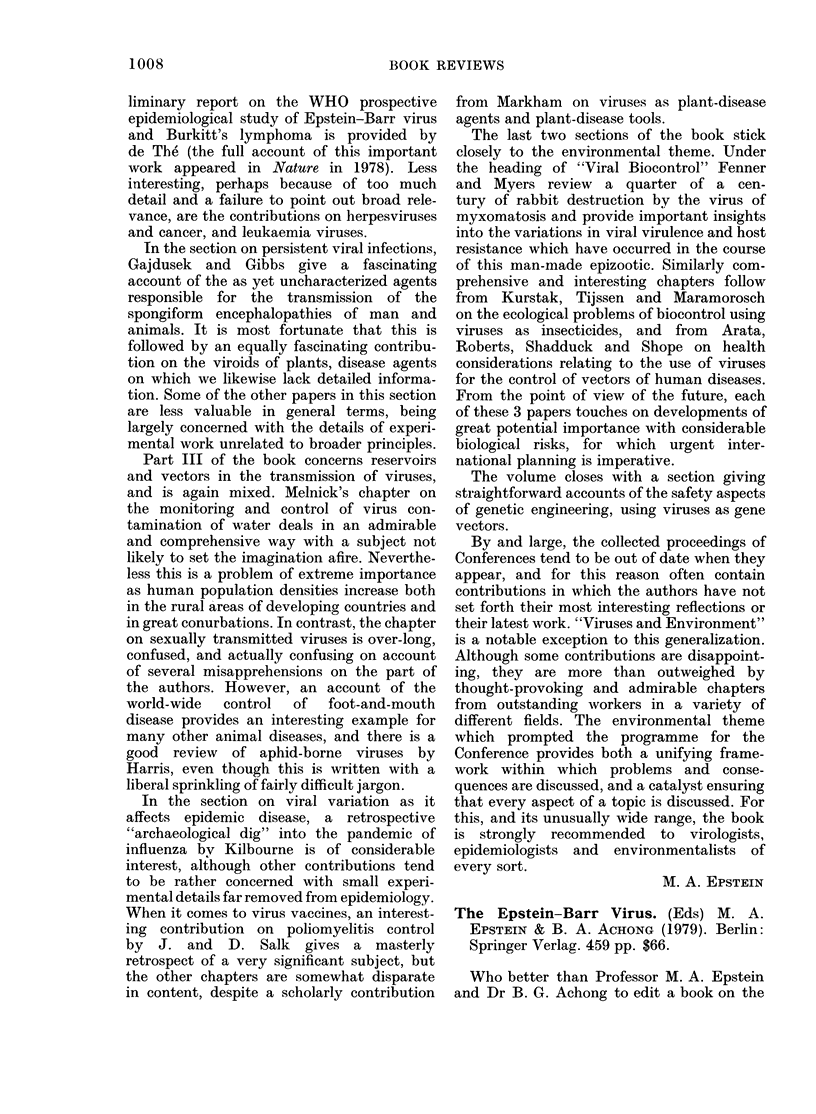# Viruses and Environment

**Published:** 1980-06

**Authors:** M. A. Epstein


**Viruses and Environment.** (Eds) E. KURSTAK & K. MARAMOROSCH (1978). New York: Academic Press. 677 pp. $32.00.

This substantial volume consists of the collected papers presented at the third in the series of International Conferences on Comparative Virology, which have been organised in Canada at 4-yearly intervals since 1969. The Conference was held in May 1977 at Mont Gabriel, Quebec, under the title "Viruses and Environment" and the present published proceedings appeared late in 1978.

In view of the origin and nature of this book, it naturally displays both the merits and disadvantages of any multi-author publication covering a wide range of subject matter. The standard of the different contributions varies considerably, not only as regards their quality and the interest of the various topics, but also in the way each is made relevant to the central theme of environmental problems. The book comprises 33 separate chapters touching on almost every area of virology, and linked together by the connecting environmental thread. Because of this topics which are not usually found as neighbours rub shoulders together in a rather stimulating and novel way.

Different aspects of virology in relation to the environment are examined in separate sections. Thus, Part I deals with viral oncology and the environment; Part II concerns persistent and unconventional viral infections in man, animals, insects and plants; Part III deals with reservoirs, vectors and viral transmission; Part IV is concerned with viral variation from the molecular as well as the epidemiological point of view; Part V relates to virus vaccines; Part VI considers the safety aspects of viral biocontrol; and Part VII is devoted to the safety of viral genetic engineering.

Within each of these different sections of the book, the views of plant virologists, animal virologists, insect virologists, epidemiologists, public health workers and molecular biologists are neatly dove-tailed, and readers from any of these disciplines will find the contributions from other fields stimulating, interesting and absorbing when set out in relation to their own more familiar work.

The overall standard of the contributions is, on the whole, very high. Professor George Klein, in the very first paper, gives an excellent and thought-provoking review of the Epstein–Barr virus in relation to human neoplasia which cannot be faulted, but which, at the same time, has little to do with the title allocated to him as an introduction to the volume—"Cancer, viruses and environmental factors". Within this section a preliminary report on the WHO prospective epidemiological study of Epstein–Barr virus and Burkitt's lymphoma is provided by de Thé (the full account of this important work appeared in *Nature* in 1978). Less interesting, perhaps because of too much detail and a failure to point out broad relevance, are the contributions on herpesviruses and cancer, and leukaemia viruses.

In the section on persistent viral infections, Gajdusek and Gibbs give a fascinating account of the as yet uncharacterized agents responsible for the transmission of the spongiform encephalopathies of man and animals. It is most fortunate that this is followed by an equally fascinating contribution on the viroids of plants, disease agents on which we likewise lack detailed information. Some of the other papers in this section are less valuable in general terms, being largely concerned with the details of experimental work unrelated to broader principles.

Part III of the book concerns reservoirs and vectors in the transmission of viruses, and is again mixed. Melnick's chapter on the monitoring and control of virus contamination of water deals in an admirable and comprehensive way with a subject not likely to set the imagination afire. Nevertheless this is a problem of extreme importance as human population densities increase both in the rural areas of developing countries and in great conurbations. In contrast, the chapter on sexually transmitted viruses is over-long, confused, and actually confusing on account of several misapprehensions on the part of the authors. However, an account of the world-wide control of foot-and-mouth disease provides an interesting example for many other animal diseases, and there is a good review of aphid-borne viruses by Harris, even though this is written with a liberal sprinkling of fairly difficult jargon.

In the section on viral variation as it affects epidemic disease, a retrospective "archaeological dig" into the pandemic of influenza by Kilbourne is of considerable interest, although other contributions tend to be rather concerned with small experimental details far removed from epidemiology. When it comes to virus vaccines, an interesting contribution on poliomyelitis control by J. and D. Salk gives a masterly retrospect of a very significant subject, but the other chapters are somewhat disparate in content, despite a scholarly contribution from Markham on viruses as plant-disease agents and plant-disease tools.

The last two sections of the book stick closely to the environmental theme. Under the heading of "Viral Biocontrol" Fenner and Myers review a quarter of a century of rabbit destruction by the virus of myxomatosis and provide important insights into the variations in viral virulence and host resistance which have occurred in the course of this man-made epizootic. Similarly comprehensive and interesting chapters follow from Kurstak, Tijssen and Maramorosch on the ecological problems of biocontrol using viruses as insecticides, and from Arata, Roberts, Shadduck and Shope on health considerations relating to the use of viruses for the control of vectors of human diseases. From the point of view of the future, each of these 3 papers touches on developments of great potential importance with considerable biological risks, for which urgent international planning is imperative.

The volume closes with a section giving straightforward accounts of the safety aspects of genetic engineering, using viruses as gene vectors.

By and large, the collected proceedings of Conferences tend to be out of date when they appear, and for this reason often contain contributions in which the authors have not set forth their most interesting reflections or their latest work. "Viruses and Environment" is a notable exception to this generalization. Although some contributions are disappointing, they are more than outweighed by thought-provoking and admirable chapters from outstanding workers in a variety of different fields. The environmental theme which prompted the programme for the Conference provides both a unifying framework within which problems and consequences are discussed, and a catalyst ensuring that every aspect of a topic is discussed. For this, and its unusually wide range, the book is strongly recommended to virologists, epidemiologists and environmentalists of every sort.

M. A. Epstein